# 

**Published:** 1906-04-07

**Authors:** 


### BOOKS RECEIVED.

J. M. Dent and Co.

"Food in Health and Disease," by H. Drinkwater.

W. Heinemann.

"The Jungle," by Upton Sinclair.

H. J. Glaisher.

"Anæsthetics for Nose and Throat," by A. De Prenderville, M.R.C.S.

J. Wright and Co., Bristol.

"The Medical Annual, 1906."

T. Nelson and Son.

"Oliver Twist."

"Kenilworth."

### THE HOSPITAL LIBRARY AND CHARITIES BUREAU.

This institution has been founded as a centre of reference in all matters concerning the establishment and management of charities. It is open to all interested in the subject on payment of a small annual fee, and inquiries by members can be made by post. Write for prospectus to the Librarian, The Hospital Buildings, 28 & 29 Southampton Street, Strand.

Inquiries of the Librarian are answered in this column free of charge. Inquiries to be answered promptly by post must be accompanied by a fee of 2s. 6d.

### MEDICAL APPOINTMENTS AND VACANCIES.

### VACANCIES.

Aberdeen (New) Poorhouse.—Resident Assistant Medical Officer.

Bridgwater Hospital.—House Surgeon.

Bristol Eye Hospital.—House Surgeon.

Canterbury, Kent and Canterbury Hospital.—House Physician.

Carlisle Non-Provident Dispensary.—Resident Medical Officer.

Central London Throat and Ear Hospital, Gray's Inn Road, W.C.—Honorary Registrar.

Chester General Infirmary.—House Physician.

Cotford, Taunton, Somerset, and Bath Asylum.—Assistant Medical Officer.

Gartloch Asylum, Gartcosh.—Assistant Medical Officer.

Hull Royal Infirmary.—Honorary Assistant Ophthalmic Surgeon.

Leeds Public Dispensary.—Junior Resident Medical Officer.

Liverpool Hospital for Consumption.—Two Honorary Assistant Physicians.

Miller Hospital and Royal Kent Dispensary, Greenwich Road, S.E.—Honorary Medical Officer for x-ray and Electro-Therapeutical Department.

National Hospital for the Relief and Cure of the Paralysed and Epileptic, Queen Square, Bloomsbury.—Assistant Surgeon.

Paddington Green Children's Hospital, London, W.—House Physician, also House Surgeon.

Reigate Town Council and Rural District Council.—Medical Officer of Health.

Royal Ear Hospital, Dean Street, Soho, W.—House Surgeon, non-resident. Also Clinical Assistants.

Royal Free Hospital, Gray's Inn Road, W.C.—House Physician and Casualty House Surgeon. Also Resident House Physician and Resident House Surgeon (Female).

Royal Halifax Infirmary.—Second House Surgeon.

Royal Hospital for Diseases of the Chest, City Road, E.C.—Resident Medical Officer. Also Clinical Assistants.

St. Bartholomew's Hospital.—Lectureship on Chemistry.

St. George's Union Infirmary, Fulham Road, S.W.—Second Assistant Medical Officer.

Tottenham Hospital, London, N.—House Surgeon, House Physician, Casualty Officer.

### IMPORTANT NOTICE.

**On account of the Easter Holidays, we beg to announce that we shall go to press with our issue of the 14th APRIL on TUESDAY NIGHT, the 10th inst. Advertisements intended for insertion in this issue should therefore reach us by 5 p.m. ON TUESDAY, the 10th inst.**

### NOTICE TO CORRESPONDENTS.

All MSS., Letters, Books for Review, and other matters intended for the Editor should be addressed The Editor, "The Hospital," The Hospital Buildings, 28 & 29 Southampton Street, Strand, W.C.

The Editor cannot undertake to return rejected MS., even when accompanied by stamped directed envelope.

### Notice to Advertisers and Subscribers.

All Advertisements, Orders for Copies of The Hospital, and Business Communications should be addressed to **THE MANAGER (Not to the Editor),** The Hospital, 28 & 29 Southampton Street, Strand London, W.C.

The Cost of Subscription, post free, is as follows:—Per week, 3½d.; per quarter, 3s. 3d.; per half-year, 6s. 6d.; per annum, 13s. Foreign and Colonial:—Per annum, 19s. 6d., payable, in all cases, in advance.

**NOTICE.—Vol. XXXVIII. of "The Hospital" April to September 1905), in handsome cloth binding, will shortly be on sale at the office of this Journal, price 10s. 6d. Cases for binding the Numbers contained in this Volume 2s. Index to Vol. XXXVIII., 3½d., post free.**

**The Monthly Part for April is now ready. Price 1s., by post, 1s. 3d.**

### A Dozen Books every Nurse should Possess.

| | | |
|---|---|---|
| 1. | **A HANDBOOK FOR NURSES.** By J. K. WATSON, M.D. Profusely Illustrated. Cloth gilt. | 5/- net, 5/4 post free. |
| 2. | **A COMPLETE HANDBOOK OF MIDWIFERY.** By J. K. WATSON, M.D. Illustrated with 143 Diagrams. Cloth gilt. | 6/- net, 6/4 post free. |
| 3. | **THE NURSES' DICTIONARY OF MEDICAL TERMS AND NURSING TREATMENT.** Compiled by HONNOR MORTEN. Fourth Edition. Suitable size for the apron pocket. Cloth. | 2/- post free. |
| 4. | **PRACTICAL GUIDE TO SURGICAL BANDAGING AND DRESSINGS.** By WM. JOHNSON SMITH, F.R.C.S. Suitable size for the apron pocket. Profusely Illustrated. Cloth. | 2/- post free. |
| 5. | **SURGICAL INSTRUMENTS AND APPLIANCES USED IN OPERATIONS.** By HAROLD BURROWS, F.R.C.S. A Classified and Illustrated List and Explanatory Notes. Cloth. | 1/6 net, 1/8 post free. |
| 6. | **NURSING HINTS TO PROBATIONERS ON PRACTICAL WORK.** By MARY ANNESLEY VOYSEY. Cloth. | 2/- net, 2/3 post free. |
| 7. | **ELEMENTARY ANATOMY AND SURGERY FOR NURSES.** By WM. McADAM ECCLES, M.S., M.B. Profusely Illustrated. Cloth. | 2/6 post free. |
| 8. | **ELEMENTARY PHYSIOLOGY FOR NURSES.** By C. F. MARSHALL, M.D., B.Sc., F.R.C.S. Profusely Illustrated. Cloth. | 2/- post free. |
| 9. | **SURGICAL WARD-WORK AND NURSING.** By ALEXANDER MILES, M.D.Edin. Second Edition. Illustrated. Cloth gilt. | 3/6 net, 3/10 post free. |
| 10. | **OPHTHALMIC NURSING.** By SYDNEY STEPHENSON, M.B., F.R.C.S.E. New Revised Edition. Illustrated. | 3/6 net, 3/10 post free. |
| 11. | **THE EXAMINATION OF THE URINE.** By J. K. WATSON, M.D. Cloth boards. Suitable size for the apron pocket. | 1/- net, 1/1 post free. |
| 12. | **LECTURES ON NURSING OF INFECTIOUS DISEASES.** By Dr. J. WOOLLACOTT. Cloth. | 2/6 net, 2/9 post free. |

SEND FOR LATEST CATALOGUE OF NURSING MANUALS.

*The above Books are obtainable of all Booksellers, or of*

**THE SCIENTIFIC PRESS, Ltd.,** 28 & 29 SOUTHAMPTON STREET, STRAND, LONDON, W.C.

